# Tomatoes: An Extensive Review of the Associated Health Impacts of Tomatoes and Factors That Can Affect Their Cultivation

**DOI:** 10.3390/biology11020239

**Published:** 2022-02-04

**Authors:** Edward J. Collins, Cressida Bowyer, Audrey Tsouza, Mridula Chopra

**Affiliations:** 1 IBBS, School of Pharmacy and Biomedical Sciences, University of Portsmouth, Portsmouth PO1 2DT, UK; edward.collins3@myport.ac.uk (E.J.C.); audrey.tsouza@myport.ac.uk (A.T.); 2 Faculty of Creative and Cultural Industries, School of Art, Design and performance, University of Portsmouth, Portsmouth PO1 2UP, UK; cressida.bowyer@port.ac.uk

**Keywords:** tomato, lycopene, vitamin C, horticulture, cardiovascular, neurodegenerative, diabetes, microbiome, fertility, skin health, exercise

## Abstract

**Simple Summary:**

The research outlined in this review paper discusses potential health benefits associated with a diet enriched with tomatoes and tomato products. This includes details of previous studies investigating the anticancer properties of tomatoes, protection against cardiovascular and neurodegenerative diseases and diabetes, maintenance of a healthy gut microbiome, and improved skin health, fertility, immune response, and exercise recovery. The specific parts of a tomato fruit that contribute these health benefits are also outlined. The potential disadvantages to a tomato-rich diet are detailed, especially the consumption of supplements that contain compounds found in tomatoes, such as lycopene. This review also discusses how the cultivation of tomato plants can affect the nutritional value of the fruit harvested. Different environmental growing conditions such as light intensity, growing media, and temperature are explained in terms of the impact they have on the quality of fruit, its nutrient content, and hence the potential health benefits acquired from eating the fruit.

**Abstract:**

This review outlines the health benefits associated with the regular consumption of tomatoes and tomato products. The first section provides a detailed account of the horticultural techniques that can impact the quality of the fruit and its nutritional properties, including water availability, light intensity, temperature, and growing media. The next section provides information on the components of tomato that are likely to contribute to its health effects. The review then details some of the health benefits associated with tomato consumption, including anticancer properties, cardiovascular and neurodegenerative diseases and skin health. This review also discusses the impact tomatoes can have on the gut microbiome and associated health benefits, including reducing the risk of inflammatory bowel diseases. Other health benefits of eating tomatoes are also discussed in relation to effects on diabetes, the immune response, exercise recovery, and fertility. Finally, this review also addresses the negative effects that can occur as a result of overconsumption of tomato products and lycopene supplements.

## 1. Introduction

Tomatoes (*Solanum lycopersicum*) are a good source of phytochemicals and nutrients such as lycopene, potassium, iron, folate, and vitamin C [1,2]. Besides lycopene and vitamin C, tomatoes provide other antioxidants, such as beta-carotene, and phenolic compounds, such as flavonoids, hydroxycinnamic acid, chlorogenic, homovanillic acid, and ferulic acid [1,2,3].

Tomatoes can make an important contribution to a healthy diet and can be consumed raw or cooked while still maintaining their nutritive value [1]. Over 80% of all commercially grown tomatoes are consumed as processed products such as juice, soup, and ketchup [4]. A diet rich in tomatoes and tomato products is known to offer several health benefits and many of these benefits are attributed to their antioxidant content [1,5,6]. This review will discuss the impact of growing conditions on the tomato cultivar as well as their health-related properties.

The potential health benefits of tomatoes discussed in this review include anticancer properties of lycopene in relation to its anti-angiogenic properties, the reduction in insulin-like growth factor (IGF) in blood, and the modulation of cellular pathways that lead to cancer. Anticancer properties of other components of tomatoes, including its fibre, vitamin C, and phenolic constituent ferulic acid, have also been discussed. Tomatoes may also help to reduce the risk of cardiovascular disease, with studies showing associations between tomato consumption and a reduction in hypertension and the risk of atherosclerosis. Observational and experimental studies highlighting neuroprotection and a role in diabetes-associated oxidative stress have been mentioned. The association between tomato consumption and skin health, in particular the protection against atopic dermatitis, is discussed. This is followed by the impact tomatoes have on the gut microbiome and how this may lead to a reduced risk of liver inflammatory disease and inflammatory bowel diseases. Further potential health benefits of tomatoes are then discussed, such as improved exercise recovery and decreased muscle damage after physical exertion, immune system modulation, and reduced risk of infertility.

## 2. Factors Affecting Tomato Crop Cultivation and Its Nutritional Value

Tomato cultivation is a major industry, and global production in 2018 was estimated at 182 million tons [7] in 2018, rising to 186 million tons in 2020 [8]. It is known that growing conditions such as water availability can impact the growth, metabolism, and yield of plants [9]. The key limiting factors to consider in crop growth are water availability, temperature, salinity, and contaminants [10]. Greenhouse systems allow control over many factors in tomato cultivation, including light intensity, temperature, and humidity [11].

Water availability affects plant growth, rate of photosynthesis, fruit production, and quality of tomato crop [11,12]. Due to this, the use of plant fertigation in combination with a drip irrigation system is becoming increasingly common in tomato cultivation [13]. These systems are beneficial not only for the regular and reliable watering of tomatoes but also for the application of a controlled dosage of fertiliser added at regulated times in the growth stage [13].

A 2010 study focusing on the effects of drought in tomato plants grew five varieties of cherry tomato plants—Kosaco, Josefina, Katalina, Salome, and Zarina—and subjected them to 50% of a standard watering regime compared to a control with 100% [12]. This study found that the Zarina cultivar was the most tolerant to stress, showing greater biomass, leaf relative water content, relative growth rate, and a higher antioxidant activity [12]. The authors concluded that tomato plants show genotypic differences to oxidative stress caused by drought and suggested that the Zarina cultivar be used in any studies aiming to improve the growth of drought-tolerant plants [12]. It is, however, worth noting that drought- and heat-induced stress reduces the growth and yield of tomato crops but increases their carotenoid content and antioxidant enzyme activity, likely due to raised oxidative stress induced by such conditions [14].

It has been reported that the rate of plant photosynthesis can be impacted by drought [15]. The stress created by drought may cause an energy imbalance in which the energy absorbed through photosynthesising complexes is more than photosystem II can dissipate [16]. It has been suggested that this excess energy is dissipated in cells by the conversion of O_2_ into reactive oxygen species (ROS), resulting in plants synthesising antioxidants such as superoxide dismutase [17,18,19].

The accumulation of antioxidants and phytochemicals in tomato fruit is also heavily impacted by the environmental conditions (light intensity, water availability, temperature, growing media salinity) that the fruit is grown in [20]. A 2009 trial compared tomatoes grown in Ireland with tomatoes grown in Spain to investigate if geographical location impacts carotenoid content in four different tomato types (cherry, plum, round, and on the vine) [21]. The authors concluded that the geographical location rather than the type of tomato had a bigger impact on the bioaccessibility (bioavailability after consumption of tomatoes) of carotenoids in the fruit [21]. The bioaccessibility of carotenoids such as lycopene is an important factor for the health benefits gained by eating this fruit.

Vitamin C content in fresh tomatoes increases to a maximum and then decreases during the ripening process [22]. It was reported by Abushita et al. that salad tomatoes grown in field conditions contained 15–21 mg/100 g fresh weight (FW) of vitamin C compared to a range of industrial grades of tomatoes with an average vitamin C value of 19 mg/100 g FW [23]. As vitamin C has been linked to immune modulation [24], this implies that the growing conditions of tomato fruit could impact the immune benefits associated with it.

Another factor to consider in the cultivation of tomatoes is the temperature. Higher temperatures are known to affect photosynthesis as they can cause damage to the photosynthetic apparatus, leading to the inhibition of photosystem II [25,26]. It has also been reported that high temperatures reduce photosynthesis through the inhibition of the ribulose-1,5-bisphosphate carboxylase in the Calvin cycle, leading to an inactivation of carbon dioxide (CO_2_) fixation [27]. A 2005 study focussed on the effects of high temperatures in tomato cultivation; researchers exposed a group of Campbell-28 variety plants to heat shock treatment of 45 °C for 2 h and measured gas exchange, chlorophyll fluorescence, and electrolyte leakage [25]. This study concluded that the heat shock treatment resulted in reductions in the net photosynthetic rate of the plants due to changes in the Calvin cycle and in photosystem II functioning [25].

Temperature impacts the distribution of photoassimilates (biological compounds formed by assimilation using light-dependent reactions) between the fruit and the rest of the tomato plant [28]. At higher temperatures, photoassimilate accumulation in fruits is increased, impacting vegetative growth of the tomato plant [21,29]. The temperature of the growing environment also affects water distribution in the plant, the cellular structures affecting the quality of the fruit (such as size and colour), and fruit development [21,30,31].

The type and number of phenolic compounds found in tomato fruit are known to vary greatly with plant genotype, fruit storage, and light intensity during cultivation [8,32]. A 2006 study grew two tomato cultivars under two different conditions: one designed to transmit ambient solar UV radiation in the range 290–400 nm, the other designed to block UV radiation below 380 nm [33]. The phenolic content of these tomatoes was tested using high-pressure liquid chromatography and a colorimetric Folin–Ciocalteu assay, and the results indicated that the higher wavelength and intensity of UV radiation exposure of the tomato plants during cultivation significantly increased the phenolic levels of the fruit level [33], which are beneficial to health.

The growing media used for tomato cultivation is also known to impact the growth and health of the plants and the resulting fruit. In tomato greenhouse production, soilless cultivation systems are in place using solid substrates [34] such as peat, bark, rockwool, synthetic foams, and perlite [35]. Sphagnum peat moss, harvested from wetland ecosystems, is a common growing medium in horticulture due to its high nutrient exchange capacity [36].

The physical properties of the substrates, such as pore size, tortuosity, and continuity, are determined by substrate particle size and shape and can affect the availability of water and air [37]. A study in 2004 tested seven substrates in greenhouse tomato cultivation: rockwool, fresh spruce sawdust, spruce wood shaving, composted spruce bark, fine blond peat, and mixtures of 66% fine blond peat +33% composted spruce bark and 33% fine blond peat +66% composted spruce bark [37]. Substrate performance was assessed according to water retention, hydraulic conductivity, pore tortuosity, and gas diffusivity [37]. While the physical properties of these substrates varied greatly, yield was not related to these properties, and if irrigation is adjusted for the physical properties of each substrate, then all tested substrates can be utilised for tomato greenhouse cultivation [37].

A study carried out in 2017 investigated three different growing media—rockwool, coconut coir, and peat–vermiculite to understand how they affected tomato plant growth, fruit yield, and quality [38]. Tomato plants grown with coconut coir had an increased photosynthesis rate, individual fruit weight, and total fruit yield [39]. This study observed that coconut coir significantly increased potassium and sulphur uptake compared to tomato plants grown on rockwool, and an increased phosphorus and sulphur uptake compared to peat–vermiculite growing media [38].

A 2015 study assessed the impact of growing media on the nutritional quality of tomato fruit by growing tomato plants with a compost prepared using effective microorganisms (EM)—a combination of microbial inoculants that stimulate plant growth [39]. The authors showed that the EM supplement not only improved plant growth and fruit yield but also lycopene content, antioxidant activity, and defence enzyme activity compared to the control [39].

A 2021 study compared soil-based growing media with hydroponic growing systems using rockwool with either drip-feed irrigation or deep-water culture for the cultivation of tomato plants [40]. This study observed that tomato plants grown with the two hydroponic systems were more water efficient and had a lower transpiration rate, requiring less water than tomato plants grown in soil [39]. It was also observed that the total lycopene and β-carotene fruit content was highest in the deep-water culture system [40].

In a 2020 study, 20 tomato varieties grown in medium and high levels of soil salinity were examined for their lycopene, vitamin C, total phenolic content, and total antioxidant capacity, and it was reported that tomato plants with a tolerance to higher soil salinities produce fruit with increased levels of antioxidants, such as phenolic compounds, and carotenoids, such as lycopene [41]. This suggests that the salinity of tomato plant growing media can directly impact the nutritional quality associated with the fruit, and hence the health benefits associated with this fruit.

It should be noted that inorganic substrates such as rockwool and perlite require large amounts of energy to manufacture and are not biodegradable, making them less sustainable than other substrates [34]. Peat is another substrate known to be unsustainable when utilised for crop cultivation [42]. The harvesting of peat has negative effects on wetland ecosystems, including the loss of peat bogs, which have a major role as carbon sinks [43]. Research is currently focused on the use of sustainable substrates such as wood fibres, bark, or recycled waste products from industries for the sustainable cultivation of tomatoes and other crops [42,44,45,46,47].

A sustainable growing media amendment under investigation is chitin and chitosan, which are waste products of the shellfish industry [48]. Studies have been carried out assessing the potential benefits these waste products have on the production of various crops [49,50,51,52,53]. A 2004 study observed an approximate 20% increase in yield for two out of three tomato trials with chitosan applied to soils and leaves as 2.5–5 mL/L solutions [53]. In all three tomato trials chitosan application resulted in significant control of powdery mildew, a fungi which weakens a plant and causes fruit to prematurely ripen [53].

It is common practice for tomatoes to be harvested at the mature green stage for ripening in transit [54], and this can impact the levels of antioxidants such as lycopene, which are synthesised during ripening [21]. However, unlike carotenoids such as lycopene and β-carotene, the vitamin C levels of tomatoes are reported to be lower in tomatoes picked at the fully ripened stage compared to those picked at the mature green stage and ripened off the vine [55].

In summary, the antioxidant and phytochemical content of tomatoes can be influenced by environmental conditions, including light intensity, water availability, temperature, and growing media as well as the ripeness stage, and all this can have an impact on their potential health effects.

## 3. Tomato Constituents for Health

**Figure 1:**
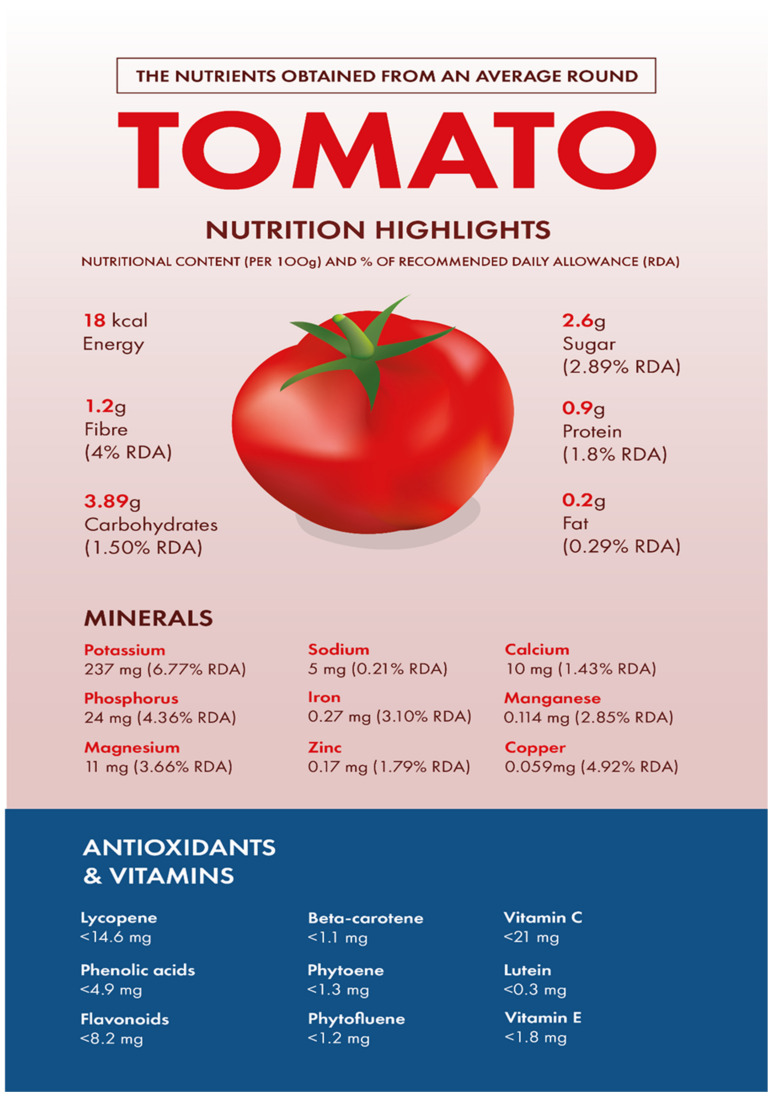
Infographic representing the nutrients obtained from an average round tomato and how these relate to the daily recommended intakes [68,69].

Tomato fruit is a fleshy berry of varying sizes and colours [56]. The fruit is composed mostly of water (>90%), with very little protein or fat, and around 3% carbohydrates (glucose and fructose) [56]. The nutrients obtained from an average round tomato and how these relate to the recommended daily intakes per person is described in Figure 1. Tomato fruit has a pericarp, which includes an outer layer of exocarp and inner layers of mesocarp and endocarp [57]. The fruit exocarp (epidermis) consists of a thin cuticle with no stomata, the phenolic content of which increases during fruit growth [57,58]. Tomato cuticle is mostly composed of a lipid polymer known as cutin, and waxes, which are complex and variable [59]. The mesocarp contains fruit vascular tissue connected to pedicel vascular tissue [57]. Vascular tissue is located in the centre of tomato fruit, supplying seeds with necessary water and minerals, and is also parallel to the fruit surface [57]. Within the unicellular endocarp boundary are seed-containing cavities derived from carpels, known as locules [57,60]. The number of locules within a fruit can vary, changing the size and shape of the fruit [60]. Locules are divided by a septum, with seeds bound to an elongated axial placenta [61]. Tomato seeds are known to contain steroidal saponins called lycoperosides, particularly lycoperoside H, which are believed to exert anti-inflammatory effects [62,63].

A study carried out by Moretti et al. analysed the chemical composition of different sections of tomatoes [64]. It was observed that vitamin C content is highest in the locule tissue (228.90 ± 5.44 mg/kg) compared to the pericarp tissue (194.90 ± 2.13 mg/kg) [64]. This study also found both total carotenoid and total chlorophyll levels to be higher in pericarp (108.03 ± 2.22 mg/kg and 0.40 ± 0.03 mg/kg, respectively) than locule tissue (87.84 ± 2.23 mg/kg and 0.33 ± 0.06 mg/kg, respectively) [64]. The presence of oxalic acid in tomatoes has been linked to renal disease, especially renal stones; however, it is worth noting that the oxalic acid content of tomatoes is reported to be between 5–11 mg per 100 g FW [65]. The oxalic acid content is suggested to increase with the ripeness of the fruit [66], and one of the suggested mechanisms for this increase is due to the conversion of ascorbic acid to oxalic acid as the fruit ripens. Cooking tomatoes, especially boiling fresh tomatoes, has been suggested to reduce their oxalic acid content [67].

The health-beneficial properties of tomatoes are studied the most in relation to their role in cancer prevention. Not only cancer but several other age-related diseases such as cardiovascular, diabetes, and Alzheimer’s as well as skin health, fertility, and exercise recovery can be influenced by constituents of tomatoes. There are several reviews that have addressed anticancer and cardioprotective properties of tomatoes but most of them focused these effects on the lycopene constituent. Tomatoes have a range of other nutrients that could confer their biological properties, as shown in Table 1. The aim of this review is to provide comprehensive literature on the health-related properties of tomatoes that can be attributed not just to lycopene but also to their other constituents.

## 4. Health Effects

### 4.1. Tomatoes and Cancer Pathology

Cancer is a leading cause of death worldwide, accounting for nearly 10 million deaths in 2020 [142]. Schwingshackl et al. discussed the effects of a tomato-rich Mediterranean diet on the risk of overall cancer mortality [143]. This paper observed that, in a clinical trial, a Mediterranean diet was found to reduce cancer incidence by 61% and also stated that a “healthy diet” can prevent approximately 30% of cancers [143]. A review by Farinetti et al. studied the benefits of the Mediterranean diet on colorectal cancer, with lycopene in particular as an important component of this diet, including polyphenols from olive oil and red wine resveratrol, which act to inhibit molecular cancer pathways in vitro [119]. The health benefits from tomatoes are enhanced as part of the Mediterranean diet as lycopene is more readily absorbed in the intestines when it has been dissolved in olive oil and heated [119].

Lycopene and β-carotene are two important carotenoids found in tomatoes and both have been suggested to confer the anticancer properties of the fruit. Lycopene, a red pigment found in tomatoes and tomato products, has antioxidant and free radical scavenging activity, and is known to be the most effective singlet oxygen quencher among the natural carotenoids [5,95,144]. The human body absorbs a significant proportion (23–24%) of ingested lycopene that proceeds to circulate and accumulate in blood plasma, liver, and other tissues with a half-life of 12–33 days [145]. Among the various plausible beneficial effects of lycopene, its anticancer properties have been studied the most. These suggestions initially stemmed from epidemiological [146,147,148,149] studies and were later supported by several experimental studies [77,78,150,151,152,153,154,155,156,157,158]. Various anticancer mechanisms of lycopene include the modulation of gene functions and apoptosis, increasing gap junction communications, anti-angiogenic effects [146,150,159], and antioxidant, anti-inflammatory, and anti-lipid peroxidation activities [160,161,162,163].

Due to their antioxidant properties, lycopene and other carotenoids are suggested to protect against carcinogenesis by preventing oxidative damage in DNA and proteins through antioxidant mechanisms [164]. It has been observed that the cleavage of lycopene via in vitro oxidation at random conjugated double bonds in the molecule forms monocarbonyl compounds [151,165]. Zhang et al. [151] showed that the products of lycopene oxidation can induce apoptosis in cancer cells. This was further investigated by Arathi et al. [152] who extracted and autoxidised lycopene from ripened tomatoes and used the products in in vitro cell culture assays to assess the toxicity and apoptosis-inducing ability in various cancer cells. This study found that there were several unknown metabolites or oxidation products of lycopene that may be involved in the inhibition of cancer cell proliferation through modulating cell cycle progression [152]. This study also demonstrated that chemically induced lycopene oxidation products were a key component in the induction of apoptosis in cancer cells [152].

A review published in 2020 by Przybylska discussed the anticancer properties of lycopene, particularly in prostate cancer [153]. This paper evaluated lycopene’s effects on prostate cancer, discussed in later sections of this review, as well as breast cancer, the second most prevalent cancer in the world [153]. Przybylska states that lycopene consumption can reduce the blood concentration of insulin-like growth factor 1 (IGF-1) via the stimulation of synthesis of a protein that binds IGF-1 [153]. It has been shown that IGF-1 is an important factor in the development of breast cancer in pre-menopausal women, and therefore lycopene’s reduction in this growth factor may reduce the risk of this cancer [154]. This paper further discusses how lycopene inhibits the proliferation of oestrogen-dependent/-independent cancer cells through multiple mechanisms, including inhibiting the activation of genes responsible for the cell cycle or protein-1-responsive genes [153,166].

Another 2020 paper by Saini et al. reviewed the anticancer properties of lycopene [78] and concluded that the antioxidant abilities of lycopene via a reduction in ROS in cells play a key role in the anticancer properties of this carotenoid.

The phosphoinositide 3-kinase/protein kinase B (PI3K/AKT) pathway has been of interest in cancer biology for decades [155]. Mutations or aberrations to this pathway are found in many cancers, and the inhibition of PI3K presents a therapeutic target for a range of tumour types [156]. AKT is known to promote cell growth and survival and is further upregulated in breast, prostate, and other forms of cancer [156]. AKT plays a part in tumour-induced angiogenesis as AKT is activated downstream of vascular endothelial growth factor (VEGF), promoting cell growth and angiogenesis, which is critical for the survival of tumour cells [155,159].

A study by Tang et al. [157] investigated the inhibitory effects of lycopene on the AKT signalling pathway in HT-29 human colon cancer cells [157]. It was observed that the proliferation of HT-29 colon cancer cells was inhibited by lycopene in a dose-dependent manner. This study concluded that lycopene treatments may inhibit the PI3K–AKT pathway and further demonstrated the involvement of this pathway in tumour development [157].

Downstream signalling through the PI3K–AKT pathway increases the expression of transcription factor hypoxia-inducible factor-1 (HIF-1) which upregulates the expression of VEGF [155]. Therefore, it can be speculated that the suppression of this pathway could prevent tumour development [167,168].

VEGF is also the fundamental regulator in cellular signalling of angiogenesis, which supplies tumour cells with blood supply [169]. Two studies using human umbilical vein endothelial cells (HUVEC) demonstrated anti-angiogenic effects of lycopene, and one of these studies showed that lycopene also inhibited angiogenesis in freshly dissected rat aorta cells at physiologically relevant concentrations of 1–2 μmol/L [159]. In another study, lycopene was shown to inhibit angiogenesis both in vitro and in vivo by inhibiting the MMP-2/uPA system through VEGFR2-mediated PI3K–AKT and ERK/p38 signalling pathways [168]. A prospective study highlighted that angiogenic potential, a biomarker of lethal cancer, was lower in individuals who had been consuming tomato products for a longer period of time [169].

The PI3K–AKT pathway also activates oncogenic signalling pathways via the transcription factor nuclear factor kappa-light-chain-enhancer of activated B cells (NF-κB) and Wnt/β-catenin [169]. NF-κB influences cell growth, proliferation, and metabolism [170] and is known to play a key role in the development of cancers [171]. NF-κB dimers are pro-survival transcription factors and are usually cytoplasmic due to interactions with the inhibitors of kappa B (IkBs); they therefore remain transcriptionally inactive [171,172]. NF-κB activation may result from different signalling pathways triggered by a variety of cytokines, or growth factors, and involves the phosphorylation and proteasome-dependent degradation of IkBs [171,173]. NF-κB activation leads to nuclear translocation followed by the transcription of target genes involved in the oncogenic pathway [171].

NF-κB is known to be active in several tumour cell types, including leukaemia, breast, and prostate [174]. A study by Assar et al. [77] studied the effects that dietary lycopene would have on several points along this oncogenic pathway. This experiment examined the effects in two human cancer cell lines, prostate (PC3) and breast (MDA-MB-231), in the absence and presence of lycopene at concentrations of 0.5–5 µM [77]. This study not only conducted MTS (3-(4,5-dimethylthiazol-2-yl)-5-(3-carboxymethoxyphenyl)-2-4-sulfophenyl)-2H-tetrazolium) cell growth assay and Western blots but also NF-κB-responsive gene activation reporter assays to monitor the pathway’s activity in real-time [77]. This study concluded that lycopene inhibits the NF-κB pathway at different stages for both breast and prostate cancer cells in vitro [78]. NF-κB and the Wnt/β-catenin signalling pathways cross-regulate each other’s activities and functions.

The Wnt/β-catenin signalling pathway is involved in cell proliferation and can lead to cancer development [172]. This pathway is upregulated by inflammation and oxidative stress, which can lead to a variety of cancers [150]. Therefore, it can be suggested that a reduction in ROS caused by lycopene or other antioxidants found in tomatoes leads to the inhibition of Wnt/β-catenin signalling. The Wnt/β-catenin signalling pathway is associated with colorectal cancer [169]. A 2019 study by Kim et al. [150] explored the mechanism by which lycopene can influence cancer cell growth through the induction of apoptosis in human gastric cancer AGS cells. Various apoptotic indices such as cell viability, DNA fragmentation, and ROS concentrations were examined in the gastric cancer cells [150], and the authors concluded that lycopene at 0.3% final concentration led to the induction of apoptosis by inhibiting Wnt/β-catenin signalling, stopping the nuclear translocation of β-catenin and suppressing the expression of specific cell survival genes.

Furthermore, a study by Preet et al. [158] tested the effect of lycopene on human breast cancer cell lines by measuring protein compounds associated with the Wnt/β-catenin signalling pathway and cancer cell viability. Preet et al. [158] showed that lycopene treatment in combination with quinacrine (a derivative of the naturally occurring compound quinine) inhibited the proliferation of breast cancer cells. It was concluded that the reduced proliferation of the breast cancer cells was a result of the inhibition of the Wnt/β-catenin signalling pathway [158].

Lycopene is the key antioxidant found in tomatoes and is the focus of many cancer studies. However, tomatoes also contain β-carotene. β-Carotene is a provitamin and is converted into retinol—a compound needed for vision [9]; it has been the focus of many studies that conclude that it is associated with anticancer activities, including inducing cancer cell apoptosis and reducing cancer cell proliferation [174,175]. Tomatoes also contain a diverse array of other potentially chemo-preventive compounds that are not the primary focus of current research, including vitamins and phenolic constituents [176].

For example, vitamin C is thought to reduce the risk of stomach carcinogenesis by controlling levels of ROS that can lead to DNA damage, or by stopping the development of carcinogenic nitrosamines introduced as part of the diet [133,177]. The effectiveness of vitamin C as an anticancer agent was debated until a 2011 study investigated the impacts of vitamin C on the human body, which concluded that, when ingested, vitamin C blood concentrations are highly controlled by renal reabsorption [178]. It was concluded that at a pharmacological dose administered intravenously, the blood plasma levels of this nutrient can be raised to 25–30 mmol/L, a concentration that has been shown to be cytotoxic to cancer cells [178].

Ferulic acid, a phenolic acid found in tomatoes, is an effective antioxidant and is suggested to have anticancer properties [112,113]. One study investigated the effects of 24 h treatment of Caco-2 colon cancer cells with 150 µmol/L ferulic acid and found that 517 genes were significantly affected [114]. The treatment delayed cell cycle progression in the S phase via the upregulation of genes involved in centrosome assembly and the S phase checkpoint [114].

Tomato peel and seeds are composed of 60% dietary fibre [179]. When fibre is metabolised by intestinal microbiota to form short-chain fatty acids such as butyric and acetic acids, cancerous colonocytes cannot use these components as a source of energy and they accumulate, inhibiting the action of histone deacetylases in these cells [180,181]. As a result, the epigenetic regulation of gene expression in these cells is changed, reducing cell proliferation and increasing apoptosis [181].

It can be concluded that a tomato-rich diet could increase human blood lycopene levels, and this has many potential anticancer properties (Table 2). However, some healthcare professionals argue that lycopene may not be the only cancer lowering constituent of tomatoes, and perhaps it is a biomarker of tomatoes that, due to an array of constituents, confer anticancer properties [78,182].

### 4.2. Tomato’s Specific Influence on Prostate Cancer

Prostate cancer is the second most common cancer found in men worldwide [183,184]. A study by Giovannucci et al. [185] investigated dietary carotenoids and prostate cancer risk. Questionnaires were used to find trends between diet and the risk of prostate cancer, and it was found that the only carotenoid associated with a decreased risk of prostate cancer was lycopene [185]. Of the four tomato-based items high in lycopene that were listed (tomato sauce, tomatoes, tomato juice, and pizza), all except tomato juice were associated with a significantly lower risk of prostate cancer [185]. More recently, a 2018 review by Rowles et al. compared the results from 30 different articles discussing tomato consumption and prostate cancer [183]. This review concluded that there was a significant inverse association between tomato consumption and the risk of prostate cancer [183].

In tomato-rich diets, lycopene is one of the most abundant carotenoids found to be accumulated in blood and tissues, reaching plasma concentrations of up to 1.8 µmol/L [184,186]. Lycopene has been shown to accumulate in several tissues, including the liver and the prostate [76,184]. A review by Rao and Agarwal in 1999 compared lycopene accumulation in major organs and found that the prostate accumulated 0.8 nmol lycopene/g tissue, the adrenal glands 1.9–21.60 nmol/g, and the testes 4.34–21.36 nmol/g [5].

In 2002, a study was conducted on 60 men with adenocarcinoma of the prostate (clinical stages T1 or T2) in which their diet was supplemented with lycopene-rich pasta sauces and other meals rich in lycopene for a three-week period [187]. Blood samples showed increased serum lycopene, from baseline 0.638 μM to 1.258 μM, and increased prostate lycopene, from 0.279 nmol/g tissue prior to the trial to 0.82 ± 0.119 nmol/g after the intervention [187]. However, this study does conclude that the impact of this uptake of lycopene on prostate cells needs further research [187].

The level of insulin-like growth factor 1 (IGF-1) in the human body is associated with prostate cancer due to the mitogenic and antiapoptotic effects on prostate epithelial cells [188,189,190]. Diet is known to influence the level of IGF-1 in the human body [189,190]. Diets primarily containing red meats and dairy products were shown to increase the levels of IGF-1, whereas diets containing high amounts of fruits and vegetables, particularly tomato-containing products, were found to associate with lower levels of IGF-1 [189,190,191]. However, studies by Chan [192] and Graydon [193] found that lycopene supplementation had no significant effect on the IGF-1 levels of male subjects with and without prostate cancer.

In 2019, Applegate, Rowles, and Erdman carried out a systematic review on the impact lycopene has on prostate cancer [194]. This was focused on androgen activity, which is associated with prostate cancer growth as androgen-regulated, prostate-specific antigen (PSA) is higher in serum samples taken from men diagnosed with prostate cancer [194,195,196]. The review suggested that lycopene reduced androgen metabolism and signalling, one of the main factors influencing prostate cancer growth and progression [194].

Obermüller-Jevic et al. [197] observed that human prostate epithelial cells treated with 5 μmol/L lycopene showed no expression of cyclin D1. A similar effect on cell growth inhibition was observed in human breast and endometrial cancer cell lines with lycopene. Cyclin D1 is a regulatory subunit of cyclin-dependent kinases CDK4 and CDK 6, which allows cells to transit from the G1 phase of the cell cycle to the S phase and is synthesised in the G1 phase that accumulates in the nucleus [197,198,199,200]. Wertz et al. [198] provided a detailed review of the mode of action of lycopene and highlighted that the inhibition of cell growth by lycopene involves the downregulation of cyclin D1, but not of cyclin E, and leads to cell cycle arrest at the G0/G1 phase. In the absence of functioning cyclin D1 in the G1 phase, cell cycle progression is halted, and the cell proliferation rate is reduced [199].

Gap junctions are intracellular channels formed by connexin proteins, joining cells and allowing the passage of nutrients and intracellular signalling molecules [201]. In a healthy prostate, basal cells use connexin 43 gap junctions in communication, and luminal cells use connexin 32 gap junctions [201]. It has been reported that in differentiated prostate cancer there is decreased expression of both channels [201]. Overall, lycopene treatments have shown an upregulation of connexin 43 expression and enhanced the gap junction channel communication in mouse fibroblast cells and prostate gland cells [198]. Through the upregulation of connexin 43 and an increased gap junction channel communication, lycopene inhibits carcinogen-induced neoplastic transformation in cell culture [198,201].

This review details the potential anticancer properties associated with the consumption of tomatoes. Most research focuses on lycopene as the primary anticancer agent in tomatoes (Table 2). Lycopene is also the focus of many published reviews that do not discuss other naturally occurring tomato compounds with anticancer associations [77,152,202]. This review, however, details not only the anticancer properties of lycopene but also vitamin C, β-carotene, ferulic acid, and the dietary fibre incorporated in tomato tissues. This highlights the importance of how the suggested anticancer properties associated with tomatoes may not derive solely from lycopene but from a combination of anticancer compounds naturally occurring in this fruit.

**Table 2:** Main findings of the effect of tomato compounds on cancers.

Biological Property Studied	Type of Study (In Vitro/In Vivo)	Main Findings	References
Antioxidant and anticancer activity	In vitro study with human prostate cancer (PC-3) and human breast adenocarcinoma (MCF-7) cell lines.	Cell viability assay showed chemically induced lycopene oxidised products (1–50 µM) were a key component in cancer cell apoptosis.	[152]
	In vitro study with HL-60 human promyelocytic leukaemia cells.	Products of lycopene oxidation, identified by spectral analyses, were added to HL-60 cell suspension as a 1% (*v*/*v*) concentration. This treatment was shown to induce apoptosis in leukaemia cells, shown using flow cytometry to evaluate the ratio of apoptotic cell death.	[151]
Anti-angiogenic role in cancer cells	In vitro study testing human umbilical vein endothelial cells (HUVEC) and rat aortic rings.	Lycopene inhibited angiogenesis in HUVEC and rat aortic rings at physiologically relevant concentrations (1–2 μmol/L) when angiogenesis was analysed using phase-contrast microscopy.	[159]
	In vitro and in vivo study testing human umbilical vein endothelial cells (HUVEC).	Lycopene (0, 1, 5, 10 µM) was shown to inhibit angiogenesis of HUVEC cells in vitro and in vivo by inhibiting MMP-2/uPA system through VEGFR2-mediated PI3K–Akt and ERK/p38 signalling pathways. Cell proliferation assessed using 3-(4,5-dimethylthiazol-2-yl)-2,5-diphenyl-2H-tetrazolium bromide (MTT) assay, cell migration assessed with Millipore QCM™ Endothelial Migration Assay Kit.	[168]
	Longitudinal cohort study.	Lycopene used as a marker of tomato intake and higher intake inversely correlated with total, and the aggressive nature of prostate cancer. The reduced severity of cancer and lesser degree of angiogenesis were reported only in individuals who consumed a tomato-rich diet for a long time period but not in those whose intake recently increased. Tissue microarrays and immunohistochemistry were used to assess tumour biomarker expression.	[169]
Modulation of molecular pathways in cancer cells	In vitro study with HT-29 human colon cancer cells.	Lycopene treatment (0, 2, 5, 10 µM) was shown to inhibit the PI3K–AKT signalling pathway in colon cancer cells, demonstrating its effects on tumour development via angiogenesis inhibition. Assessment of cell proliferation using MTT assay and gene expression investigated using transient transfection and luciferase reporter assays.	[157]
	Ex vivo and in vivo study testing human umbilical vein endothelial cells (HUVEC) and rat aortic rings.	Lycopene (400 μg/mouse) reduced angiogenesis cell signalling through inhibition of the VEGF cell signalling pathway. Anti-angiogenic activity of lycopene confirmed by ex vivo rat aortic ring and in vivo chorioallantoic membrane assays.	[167]
	In vitro study with human prostate (PC-3) and breast (MDA-MB-231) cancer cell lines.	Lycopene (0.5–5 µM) inhibited different stages of the NF-κB cell signalling pathway in both cancer cell lines in vitro as seen in Western blots and NF-κB-responsive gene activation reporter assays.	[77]
	In vitro study in human gastric cancer (AGS) cells.	Lycopene at 0.3% was shown to induce apoptosis by inhibiting Wnt/β-catenin signalling, stopping the nuclear translocation of β-catenin and suppressing the expression of specific cell survival genes AGS cells. Cell viability, DNA fragmentation, and ROS concentrations were examined in these cells.	[150]
Cytotoxicity and cancer cell growth	In vitro study testing human prostate epithelial cells (PrEC).	PrEC treated with lycopene (up to 5 μmol/L) showed no expression of cyclin D1 in vitro. This regulatory subunit of kinases essential to the cancer cell cycle, resulting in reduced cancer cell cycle progression. High-performance liquid chromatography (HPLC) analysis, a thymidine incorporation assay, and flow cytometry were carried out to assess the impact of lycopene.	[199]
	In vitro study testing human prostate (PC-3) and breast (MDA-MB-231) cancer cell lines.	PC-3 and MDA-MB-231 cancer cell lines were tested in vitro in the absence and presence of lycopene at concentrations of 0.5–5 µM. MTS cell growth assays, Western blots, and NF-κB-responsive gene activation reporter assays showed that lycopene inhibits the NF-kB pathway at different stages in both cell lines.	[77]
	In vitro study treating Caco-2 colon cancer cells.	Treatment of Caco-2 colon cancer cells with 150 μmol/L dietary fibre ferulic acid delayed cell cycle progression in the S phase. Gene expression was analysed with cDNA microarray technique.	[115]
Cancer cell apoptosis	In vitro study testing human prostate cells (PC-3).	Flow cytometry analysis showed 27–32% apoptosis in PC-3 when supplemented with (10–50 μM) β-carotene.	[174]
Gap junction communication in cancer cells	In vitro study with rat liver epithelial WB-F344 cells.	Incubation of WB-F344 cells with oxidation products of lycopene (0.2% *v*/*v*) improved the gap junction communication in dye transfer assay using microinjection of the fluorescent dye Lucifer Yellow CH.	[203]

### 4.3. Cardioprotective Effects of Tomatoes

A tomato-rich diet has been linked to a reduction in the risk of heart disease. Song et al. reviewed 14 eligible studies and found a significant inverse association between lycopene intake and coronary heart disease [204]. Another meta-analysis reviewed 25 studies and reported that high lycopene consumption and lycopene serum concentrations reduced the overall mortality by 37%, cardiovascular disease by 14%, and stroke by 23% [205].

A randomised, cross-over controlled trial in healthy participants examined the effect of a single dose of raw tomatoes, tomato sauce, or tomato sauce plus refined olive oil on biomarkers of cardiovascular disease [206]. The results showed all three interventions reduced plasma cholesterol and triglycerides and raised plasma high-density lipoprotein (HDL) cholesterol and interleukin-10 concentrations. Tomato sauce plus olive oil produced the maximum effect, likely due to the increased bioavailability of lycopene as oil is known to improve this. The authors indicated that including tomatoes as a regular part of a diet may help to prevent postprandial lipemia by reducing blood triglyceride levels, and in doing so, reduce the risk of developing atherosclerosis [206]. An increase in triglyceride levels can lead to the production of small, dense low-density lipoprotein (LDL), which is highly atherogenic [207,208]. It is worth noting that the fat-soluble pigment lycopene is released from tomato cell wall protein–carotenoid complexes during food preparation, therefore the bioavailability of lycopene is higher with cooked tomatoes and tomato products such as juices and sauces than fresh tomatoes, and daily consumption of such tomato products significantly reduces blood LDL cholesterol levels in adults [209]. In a recent cross-over study, feeding of tomato sauce from vine-ripened tomatoes at 150 mL/day for 6 weeks was compared with sterol-enriched yoghurt and both interventions reduced LDL cholesterol by 12% and 15%, respectively [210].

Heart disease is a collective term that includes hypertension and atherosclerosis. Hypertension is one of the most common chronic diseases worldwide, with accompanying risks including cardiovascular disease (CVD) and kidney disease [6]. In a study conducted by Engelhard et al. [211], patients with grade-1 hypertension were found to have significantly lower systolic and diastolic blood pressure after short-term treatment with 250 mg tomato extract Lyc-O-Mato. In a double-blind placebo study of grade-1 hypertension patients, both systolic and diastolic blood pressure were significantly lower after treatment with tomato extracts [211]. 𝛾-Aminobutyric acid (GABA), a neurotransmitter present in the sympathetic nervous system, is known to lower systolic blood pressure [212,213], and tomatoes have been shown to contain high levels of GABA [214]. GABA has been reported to lower the blood pressure of hypertensive patients but not of normotensive individuals [168]. Daily supplementation of 80 mg of GABA has been found to reduce blood pressure in adults with mild hypertension [215]. A study carried out in 2008 analysed tomato varieties and found that they had an average GABA content of 50.3 mg/100 g fresh weight [216].

Many of the antioxidants found in tomatoes, including lycopene, beta-carotene, and vitamin C, protect vascular cells and lipoproteins from oxidation and thus prevent the formation of atherosclerosis [134,217]. Low-density lipoprotein (LDL) oxidation is a well-known factor in genesis [218] and the progression of atherosclerosis, a process that leads to the narrowing of arteries due to a build-up of cholesterol in subendothelial space. Oxidised LDL is believed to be important in the formation of atherosclerosis and, therefore, vascular diseases. Oxidised LDL increases the expression of pro-inflammatory cytokines, which promote the adhesion of white blood cells to the blood vessel wall [219]. This can lead to the transmigration of the adhered cells into the innermost layer of the vessel where they are transformed into macrophages, which rapidly accumulate oxidised LDL [219]. These cells are often the origin of atherosclerotic lesions, which form in artery walls and potentially lead to coronary heart disease and heart attacks [134,219]. Chopra et al. found that increased intake of fruits and vegetables, especially red coloured ones, improves the ex vivo resistance of LDL to oxidation [220]. In another human study, a 3-week low-tomato diet followed by a 3-week high-tomato diet (400 mL tomato juice and 30 mg tomato ketchup daily) led to a reduction in LDL cholesterol levels and increased ex vivo resistance of LDL to oxidation in normocholesterolaemic participants [209]. Interestingly, a study conducted in 2000 showed that the regular intake of tomato juice is associated with an increase in blood vitamin E levels [134]. Lycopene and beta-carotene are known to more effectively inhibit LDL oxidation in the presence of vitamin E [134,209,219].

Blood platelets respond to vascular damage by binding to the subendothelial matrix, eventually leading to atherosclerotic lesions, thrombus formation, and vascular events. Platelets are therefore considered as the driving force to myocardial infarction and ischaemic stroke [221,222,223]. Tomatoes have been shown to have platelet anti-aggregatory properties. In a double-blind, randomised trial, the dietary supplementation of adults 40–70 years old, these being healthy individuals, with tomato extract was shown to reduce ex vivo platelet aggregation induced by both ADP and collagen [224]. Although initially carotenoids lycopene and beta-carotene were suggested to contribute to the anti-aggregatory properties of tomatoes, later studies suggested that the anti-platelet factor of tomatoes was due to water-soluble, heat-stable compounds that are concentrated in the jelly substance surrounding the seeds [225,226]. It has been suggested that a diet containing anti-platelet compounds such as these have the potential of reducing lipid levels and lowering blood pressure and can reduce the risk of ischaemic heart disease and strokes by up to 80% in middle-aged individuals [227,228].

Many studies have shown tomato extracts to have platelet anti-aggregatory activity in vitro and in vivo and possibly preventing thrombus formation [222,224,225,226,229,230,231,232]. A study by Zhang et al. [233] investigated the impact of water-soluble tomato concentrate (WSTC) on the platelet aggregation in Sprague Dawley rats. This study found that WSTC inhibited adenosine diphosphate (ADP)-induced platelet aggregation in vitro and ex vivo in the rats without affecting their coagulation system [233]. Platelet aggregation relies on fibrinogen binding to the calcium-dependent glycoprotein (GP) IIb/IIIa complexes found on platelets [234]. When platelets are activated by ADP, these GP IIb/IIIa complexes bind with fibrinogen, leading to many platelets assembling and connecting to the same fibrinogen strands and forming a clot [235]. Zhang et al. [233] found that WSTC increased cytoskeleton stability and led to the inhibition of platelet aggregation. There are suggestions that people should adjust their diet to reduce cardiovascular risk and prevent any potential side effects, such as headaches or dizziness, nausea, and increased bleeding, including nose bleeds, associated with anti-platelet medications [236].

Inflammation plays an important role in atherosclerosis, a process associated with lipid accumulation in the artery wall [237]. Postprandial lipemia, characterised by an increase in triglyceride-rich lipoproteins, is a condition known to trigger inflammation and atherogenesis in humans [238]. The transcription factor NF-κB, previously mentioned in relation to cancer development, is also a modulator of inflammation in the liver [169]. The liver is involved in the uptake, formation, and exportation of lipoprotein and is thus an essential component of lipid metabolism [239]. Therefore, inflammation and liver damage can have a detrimental impact on lipid metabolism in mammals. Sahin et al. [169] supplemented a rat diet with tomato powder and found that this resulted in reduced liver damage caused by age-associated inflammation and oxidative stress through the inhibition of the NF-κB pathway.

The activation of NF-κB in cultures of endothelial and smooth muscle cells with inflammatory stimuli is also suggested to have a role in atherosclerosis formation [240,241]. NF-κB activation has also been observed in previous studies [241,242] and genes expressed in atherosclerotic plaques are regulated by NF-κB [242]. There is strong evidence that a reduction in inflammation can reduce the risk of atherosclerosis caused by NF-κB activation but no research to date has investigated the impact of tomato extract on atherosclerosis caused by NF-κB activation. It is worth noting that tomato powder supplementation of rats has been shown to inhibit the NF-κB pathway in the liver of animals [169].

Vascular endothelium plays an important role in the constriction and dilation of blood vessels, and its dysfunction is suggested as an early, reversible precursor of atherosclerosis. In a randomised controlled trial in post-menopausal women, the effect of 70 g tomato puree ingestion was examined on endothelial-dependent, flow-mediated dilation (FMD) and endothelial-independent, nitro-mediated dilation of the brachial artery using high-resolution ultrasound. The effects after 24 h and 7-day intake were examined [243]. Although a significant increase in plasma lycopene was observed after 7 days, it did not affect endothelium-dependent or -independent dilation of the brachial artery. Similar findings were reported by another group, where 80 g of tomato paste puree per day for 7 days failed to affect the flow-mediated dilation of the brachial artery after a standardised fat meal, however, the incorporation of tomato paste produced a significant improvement in the haemodynamic changes, such as reduction in diastolic blood pressure, increase in brachial artery diameter, and decrease in stiffness index [244].

Overall, the potential benefits of a tomato-enriched diet are associated with lowering of blood pressure; anti-platelet, anti-inflammatory, and anti-apoptotic activity; and lipid lowering, as well as the inhibition of LDL oxidation; the latter is believed to be key player in the pathophysiology of atherosclerosis, a hallmark of cardiovascular disease.

It is worth noting that the role of a tomato-rich diet on cardiovascular disease was further evaluated in a recent meta-analysis by Rosato et al. [245]. This paper described the positive impact of the Mediterranean diet on cardiovascular disease in particular foods such as olive oil, fresh fruits and vegetables, nuts, legumes, and fish, but not red meats [245]. It is stressed that it is the combination of elements in the Mediterranean diet, including the phytochemicals found in fresh fruits and vegetables such as tomatoes, that results in these positive impacts, and these elements on their own would not have the same impact [143]. Although a tomato-enriched diet has been shown to influence several key mechanisms that are important in vascular pathology, and population-based studies show an inverse correlation between their intake and cardiovascular disease, it is unlikely to affect a multifactorial disease such as CVD when used in isolation. However, if incorporated as a component of a healthy diet, it should provide an additive effect when combined with other cardioprotective nutrients in food.

Recent studies have shown that the beneficial effects of tomato compounds are not limited to cardiovascular diseases and cancer but have also been reported in neurological disorders and diabetes mellitus (DM) [74,78,109,246,247].

### 4.4. Neurodegenerative Disorders

Neurodegenerative diseases are associated with the degeneration of the nervous system over a long period of time and, among these, Alzheimer’s, Parkinson’s, and cerebral ischaemia associated with stroke are the most common neurodegenerative diseases. Neuroinflammation, oxidative stress, and apoptosis are important hallmarks of these diseases.

The majority of cases of stroke are due to cerebral ischaemia, and population-based studies have shown a negative association between tomato, especially lycopene intake, and incidence of stroke [205,248,249]. In rats, a tomato pomace powder pre-treatment at doses of 2, 10, and 50 mg/kg protected various areas of the brain, including hippocampus, striatum, and cerebral cortex affected by experimental cerebral ischaemia induced by permanent occlusion of the middle cerebral artery [250]. The tomato pomace powder pre-treatment of animals also increased the activities of antioxidant enzymes glutathione peroxidase and superoxide dismutase and decreased the lipid peroxidation product malondialdehyde in the hippocampus and cerebral cortex area of the brain.

In Alzheimer’s disease (AD), neurofibrillary tangles (aggregates of tau protein), a reduction in neurotropic factors, and amyloid-β plaque are present. Amyloid-β accumulation can induce apoptosis both via extrinsic death receptor-mediated and intrinsic mitochondria-mediated pathways, and lycopene has been shown to inhibit these pathways. In human neuroblastoma SH-SY5Y cells, the pre-treatment of cells with 0.2 and 0.5 μmol/L lycopene for one hour, followed by 24 h stimulation with amyloid-β (20 μM), significantly inhibited apoptosis through its dose-related effects on Bax/Bcl-2 and cleavage of pro-caspase-3 [251]. In addition, lycopene pre-treatment reduced amyloid-β induced oxidative stress, mitochondrial dysfunction, and NF-κB activation in the cells. Lycopene has also been shown to improve the production of brain-derived neurotrophic factor (BDNF) during neurotoxic challenges [252]. Lycopene supplementation of male Wistar rats at a dose of 5 mL/kg body weight for 21 days reduced neuro-inflammation, oxidative damage to mitochondria, and apoptosis, and improved memory retention and restoration of BDNF level in β-A1-42 treated rats [252]. Yu et al. also provided evidence that dietary lycopene supplementation could improve cognitive performance in tau transgenic mice expressing P301L mutation [253]. Likewise, Zhao et al. demonstrated that lycopene supplementation could reduce oxidative stress and neuroinflammation and improve cognitive impairment in aged CD-1 mice [254]. A recent review provided details of in vitro and animal studies describing the neuroprotective role of lycopene and related this to its antioxidant activity, the inhibition of a redox-sensitive transcription factor NF-kB, a reduced expression of amyloid-β and its precursor, a reduction in neuroinflammation, an improvement in mitochondrial function, memory, and learning as well as a restoration of antioxidant defence [77]. Vascular dementia is associated with cerebral ischaemia and a recent study examined the effects of supplementation with lycopene at a dose of 50, 100 and 200 mg/kg body weight every other day for two months in a vascular dementia model in rats. At 100 mg/kg dose, lycopene supplementation reduced the oxidative stress, improved the antioxidant enzyme superoxide dismutase and glutathione peroxidase levels in the hippocampus and improved the learning-memory ability of animals [255].

In human studies, lower antioxidant status, especially vitamin C, lycopene, vitamin E, and antioxidant enzymes superoxide dismutase and glutathione peroxidase, and high levels of markers of oxidative stress have been reported in the plasma [256,257,258,259] as well as cerebrospinal fluid [259,260,261] of AD patients. In the Nurses Health study, participants aged ≥70 years were followed up for 4 years, and a decline in cognitive function was reported to be slower with high lycopene intake but not with vitamin C, β-carotene, and vitamin E intakes [262]. A recent study that followed up elderly patients over 5–6 years reported that plasma antioxidants such as vitamin E isomers (alpha- and gamma-tocopherol), retinol, and carotenoids were not significantly associated with a reduced risk of dementia or AD [263]. The lack of association could be related to the age group of 70–75 years that was studied. AD is a chronic disease and may have already been present in the cohort that was included in the study; therefore, the study population was not likely to be a suitable age group to examine the associations.

The prevalence rate of AD is less than 1% before 65 years of age, and it increases to 10% after 65 years of age. The brain, due to its high oxygen consumption, high polyunsaturated fatty acids, and transition metals ion content, is highly susceptible to oxidative stress, and antioxidants such as vitamin C, carotenoids, and flavonoids present in tomatoes are therefore a likely candidate for the protection offered by this fruit against neurodegenerative disease. Future follow-up studies should be conducted in 60–65-year-old individuals to confirm whether tomato or its constituents, carotenoid, vitamin C, and flavonoid, intake can slow down cognitive decline with aging.

Parkinson’s, a neurodegenerative disease, is also associated with oxidative stress and neuronal apoptosis, and its pathology is also likely to be influenced by antioxidant and anti-apoptotic dietary components. The motor disability seen in Parkinson’s is suggested to be due to the degeneration of dopaminergic neurons leading to a decrease in dopamine (DA) in the striatum. Supplementation with 20% (*w*/*w*) lyophilised tomato powder for 4 weeks before methyl-4-phenyl-1,2,3,6-tetrahydropyridine (MPTP) induced Parkinson’s disease (PD) in mice was reported to prevent a striatal decrease in the DA levels [264]. In another study in mice, 7-day pre-treatment with lycopene at doses of 5, 10, and 20 mg/kg was examined on MPTP-induced PD, and treatment was found to reduce MPTP-induced oxidative stress, apoptosis, and depletion of dopamine in the striatum [265]. Likewise, vitamin C feeding of mice at a dose of 15 mg/kg body for 3 days before intraperitoneal injection of MPTP at 20 mg/kg reduced neuroinflammation and dopaminergic neuronal degradation in the striatum and improved the locomotor inability caused by the neurotoxin [266].

Overall, pathological changes seen in AD, PD, and cerebral ischaemia have been shown to be ameliorated with lycopene and tomato extract in studies that were conducted in vitro as well as in animal studies. Few human studies in AD and PD patients have indicated a protective role; however, studies are limited and not conclusive likely due to the age group that has been studied in observational as well as intervention studies. Further human studies for AD- and PD-related investigations in the age group 60–65 years old are likely to provide a better insight into the protective role that a tomato-enriched diet may offer against these neurodegenerative diseases.

### 4.5. Diabetes

Carotenoids may play a role in reducing the risk of insulin resistance and the development of diabetes, and an inverse association has been reported between plasma β-carotene, lycopene, and glucose intolerance in newly diagnosed patients [267] and on glycated haemoglobin levels in older type 2 diabetes patients [268,269,270].

Type 2 diabetes is described as a multifactorial metabolic syndrome associated with oxidative stress, inflammation, hyperglycaemia, and hyperlipidaemia [271]. Tomato constituents have antioxidant and anti-inflammatory properties. Studies have examined hypoglycaemic, hypolipidemic, anti-inflammatory, and antioxidant effects of tomatoes, especially lycopene. In experimental diabetic rats, lycopene at a dose of 10 mg/kg/day for 28 days significantly reduced the increase in blood glucose and glycated haemoglobin (HbA1c) levels induced by streptozotocin (STZ) [272]. In another study, male albino Sprague Dawley rats were fed a high-fat diet for 4 weeks followed by intraperitoneal injection of STZ at 25 mg/kg. The effects of lycopene administration at 10 and 20 mg lycopene per kg body weight/day for 10 days was examined on the fasting blood glucose, lipids, and glycosylated haemoglobin levels, and a reversal to normality of these parameters was seen with lycopene supplementation [273]. A recent case-control study reported that lycopene intake positively correlated with peripheral antioxidant activity, antioxidant enzymes superoxide dismutase, and glutathione peroxidase levels and negatively correlated with fasting blood glucose and glycated haemoglobin (HbA1c) levels in patients with type 2 diabetes [274]. A detailed account of evidence from in vitro, animal, and human studies (cross-sectional, prospective, and two randomised controlled trials) suggesting a preventive role of lycopene and tomato-enriched diet against diabetes is provided by Zhu et al. [80].

Figueriredo et al. [275] reported that a combination of lycopene with metformin had an additive effect on improvements in postprandial blood glucose levels, dyslipidaemia, and antioxidant status. The investigation was performed in rats, and STZ-induced diabetic rats were treated with lycopene (45 mg/kg) and metformin (250 mg/kg) alone and in combination for 35 days.

There is ample evidence from animal studies suggesting a decrease in diabetes-induced hyperglycaemia, dyslipidaemia, and oxidative stress. There is, however, limited evidence from human intervention trials. Shidfar et al. [276] fed type 2 diabetic patients with 200 g raw tomatoes/day for 8 weeks. No significant effect was observed in blood glucose levels. However, there was a significant improvement in both systolic and diastolic blood pressure as well as improvements in apoprotein A-1 (ApoA-1) levels. Diabetic patients are at increased risk of cardiovascular disease and the ApoA-1 constituent protein of high-density lipoprotein is important for the anti-atherogenic properties of HDL. Bose and Agarwal [277] reported that the supplementation of diabetic patients with cooked tomatoes improved the antioxidant defence and plasma lipid peroxidation products but failed to affect the lipid profile and HbA1c levels. It is important to note that the majority of studies that show hypoglycaemic effects of lycopene were carried out using pure compounds, and concentrations were much higher than likely to be achieved by the amount of tomato products that were used by Shidfar et al. [276] and Bose and Agarwal [277] for human studies.

Zidani et al. [278] reported that 12 weeks’ supplementation of mice with 46 and 84 mg of lycopene/kg of food provided by tomato peel extract significantly reduced insulin resistance caused by the high-fat diet. Both type 2 diabetes and gestational diabetes (GD) are associated with insulin resistance. In the case of GD, it is caused by a hormonal change during pregnancy. Tomatoes have a low glycaemic index, and therefore can be considered as a potential fruit of choice for pregnant women. Very few studies have examined the association or effect of tomato-rich diets on gestational diabetes. A cross-sectional study that used food frequency questionnaires to estimate the food/nutrient intake of its participants highlighted that high lycopene protected against gestational diabetes-associated hyperglycaemia in women and suggested that intake can offer a protective effect against GD [279].

To date, anti-diabetic effects in animal studies have either tested the effects of lycopene or tomato extract. Tomatoes contain glycoalkaloid esculeoside A, and its concentration is four times higher than that of lycopene. Yang et al. [100] suggested that esculeoside A can be considered as a functional supplement for diabetes. In their study, wild-type C57BLKS mice were used and esculeoside A (100 mg/kg) administration by gavage for 56 days was found to lead to a reduction in fasting blood glucose levels and improved glucose tolerance in mice [100].

Both type 2 diabetes and gestational diabetes are on the rise and can be prevented with diet and lifestyle interventions [280,281]. It is well known that synthetic hypoglycaemic medications that are used for type 2 diabetes induce side effects. Further investigations of foods such as tomato products either on their own or in combination with other hypoglycaemic foods are warranted to confirm if the findings of animal studies can be replicated in humans, as the evidence from randomised controlled trials is limited and inconclusive at present.

### 4.6. Tomato Fruit for Skin Health

The properties of tomatoes are not limited to disease prevention. Studies have provided evidence of the beneficial effects of dietary tomato and its supplements for improved skin health [282,283,284]. The principle of oral photoprotection provided by antioxidants to prevent the harmful effects from UV radiation has gained popularity over the last decade [282,283,284]. The benefits and hazards of solar ultraviolet (UV) radiation are well documented and include the effects of solar exposure on skin cancer, malignant melanoma, immune suppression, photoaging, photosensitivity, and diseases in the eye [85,86,285,286,287]. Acute UV radiation has been linked to skin burns, oedema, abnormal pigmentation, and photokeratitis, and long-term exposure increases the risk of photoaging and malignant tumours [85,86,284,286,287]. Three types of UV rays are produced by sunlight, UVA, UVB, and UVC [86,284,288]. UVA rays have the longest wavelength, followed by UVB, while UVC rays have the shortest wavelength. UVC has the strongest mutagenicity, followed by UVB, while UVA is considered a weak mutagen [85,284,287]. However, all UVC rays are absorbed by the Earth’s ozone layer, therefore, exposure is unlikely, except through an artificial source such as a laser [85,287]. UVA rays can penetrate the dermis and the subcutaneous tissue area [84,284,287]. UVB rays can reach the epidermis and have the capacity to interact with DNA [84,284,289]. The underlying mechanism involves the production of ROS by UV radiation, which hinders DNA replication and transcription and results in destructive oxidative stress, the activation of the arachidonic acid pathway, and the mediation of inflammatory responses [85,287].

Studies by Groten et al. and Aust et al. provided evidence that carotenoid-containing supplements could significantly protect against UVB-induced erythema by reducing oxidative stress [282,288]. Baswan et al. reported similar findings regarding carotenoid supplementation against both UVB-induced erythema and UVA-induced pigmentation [289]. Grether-Beck et al. examined the capacity of carotenoids, including lycopene-rich tomato nutrient complex (TNC) and lutein, to protect against UVA/B and UVA1 radiation at a molecular level [283]. Analysis of the mRNA expression of key genes involved in solar radiation-induced skin damage, including heme-oxygenase 1 (HO1), matrix metalloproteinase 1 (MMP1), and intercellular adhesion molecule 1 (ICAM1), revealed that UVB/A and UVA1 radiation significantly upregulated steady-state levels of HO-1, ICAM-1, and MMP-1 mRNA in the skin of healthy volunteers who did not receive the supplement. Moreover, TNC and lutein treatment significantly inhibited UVB/A and UVA1 radiation-induced gene expression [283]. Calniquer et al. assessed the effect of a combination of carotenoids and polyphenols (tomato extract with rosemary extract) on the response of skin cells to UV irradiation [290]. The results demonstrated that carotenoids and polyphenols worked in synergy and that combining these compounds was more effective in balancing UV-induced skin cell damage than using them separately [290].

Vitamin C is another compound found in tomatoes that contributes to immune modulation [24]. When applied topically, it is known to be actively taken up by epidermal and dermal skin cells using sodium-dependent vitamin C transporter isoforms (SVCT1 and SVCT2) [137]. These cells are involved in the production of collagen fibres and therefore are essential to the function of the skin as a barrier against pathogens [136].

The use of natural ingredients such as tomato extract or tomato seed oil in cosmetic products for skin care and health has also received popularity over the last few years [85,86,87,121,284,287,291,292,293,294,295,296,297]. Tomato seed oil has been extensively used in the production of cosmetic and personal care products such as anti-aging serums, body butter, sunscreens, and skin lightening cream due to its high linoleic acid, lecithin, antioxidant, and natural UV protection attributes [284].

Furthermore, there is increasing evidence that a diet rich in antioxidants may significantly influence the course of certain skin diseases such as atopic dermatitis, acne, and psoriasis [285,291,298]. Multiple in vitro studies in mice have revealed that polyphenols including quercetin and gallic acid present in tomatoes may be an alternative for the development of cosmetics that could be used to treat acne vulgaris [108,299,300,301].

Atopic dermatitis (AD) is a chronic relapsing inflammatory skin disease that can affect up to 25% of children within a diverse paediatric population [302]. Symptoms can include itching and scratching, dry skin, patchy eczema, exudation, and skin thickening and discolouration [302]. Although the mechanisms of the pathogenesis of AD have not been fully elucidated, the chronically inflamed skin of patients with AD plays a key role in pathogenesis, with the overproduction of ROS and a decrease in antioxidant defence [302]. Sapuntsova et al. have reported that levels of ROS were significantly higher in the skin biopsies in AD patients compared to those of controls [303]. Lycoperoside H, an anti-inflammatory component present in the seed part of tomatoes, was shown by Takeda et al. to relieve symptoms of AD in transgenic mice expressing IL-33 driven by a keratin-14 promoter (IL33tg) [62].

Other reported benefits of tomato compounds for skin health include the protection against tick bites and heavy metal toxicity [64,121,292,296,299]. Boulanger et al. showed that natural skin repellents made from eucalyptus, tomato, and coconut can protect against tick-borne infections such as Lyme disease [63]. A study by Tito et al. demonstrated that an active ingredient derived from *Lycopersicon esculentum* tomato cultured stem cells protected skin cells against heavy metal toxicity [292]. The mechanism of action involves the preservation of nuclear DNA integrity from heavy metal damage by inducing the genes responsible for DNA repair and protection and also involves the neutralisation of the effect of heavy metals on collagen degradation by inhibiting collagenase expression and inducing the synthesis of new collagen [292]. Additionally, in a study involving Indian women, Nutrova, a blend of collagen peptides and natural antioxidants from tomatoes, green tea, and grapes, has been shown to significantly reduce wrinkles, skin roughness, and hyperpigmentation while improving skin hydration and firmness [296]. Similarly, another study of 4000 women showed that a diet reported as high in potassium and vitamins A and C correlated to fewer wrinkles in patients’ skin [291].

It can be concluded that incorporating tomatoes into a diet could have benefits to a person’s skin health. The suggested benefits include protection against UV radiation through antioxidant properties [284], treatment of skin inflammatory conditions such as AD [62], and protection against tick bites and heavy metal toxicity [63,292].

### 4.7. Tomatoes, Gut Microbiome, and Inflammation

“Microbiome” is a term used to describe a community of colonising microorganisms such as fungi, viruses, and bacteria found in a particular environment [304]. Microbial populations reside throughout the human body, including the stomach and intestines, and are increasingly described as a key link between genetic and environmental impacts that affect an individual’s health [304,305]. The gut microbiome is defined as all microorganisms found in the gastrointestinal tract consisting of bacteria, archaea, viruses, and eukaryotic microbes, and can have as many as 100 trillion cells [305,306]. These bacteria are known to vary in composition depending on the lifestyle, genetics, and diet of the host [305].

The composition of the gut microbiome is implicated in the development of liver inflammatory disease and liver cancer [307]. In a 2018 study by Xia et al. [308], mice were fed a high-fat diet (HFD) supplemented with a liver-specific carcinogen (DEN) along with a tomato powder rich in lycopene, which has previously been shown to inhibit HFD-induced liver disease [309]. The tomato powder significantly increased both diversity and richness of the gut microbiota in all mice [308]. The gut microbiome contains both Gram-positive and Gram-negative bacteria, and it has been shown that an increase in Gram-negative bacteria in the gut can lead to an increase in the level of hepatotoxic compounds, such as lipopolysaccharides (LPS) [310].

LPS are major components of the outer membrane of Gram-negative bacteria and are known to induce inflammation through induction of Toll-like receptor 4, which can lead to cell proliferation and a reduction in apoptosis [129,310]. In 2018, Xia et al. reported that feeding mice tomato powder reduced the relative abundance of the gut Gram-negative bacteria by reducing levels of gut LPS in the mice, lowering the risk of inflammation [308]. Increasing the diversity and richness of the gut microbiome through tomato powder supplementation was found by the authors to regulate inflammation, lipid metabolism, and the circadian clock in the liver [308].

Inflammatory bowel diseases (IBDs) are chronic inflammatory conditions of the gastrointestinal tract and include Crohn’s disease and ulcerative colitis [308]. Both of these conditions are affected by a number of factors, including abnormal gut microbiota [311]. A study by Scarano et al. (2018) [129] developed a bronze tomato line that was shown to have 30–50% higher levels of flavanols and anthocyanins when compared to other tomato lines. These tomatoes were freeze-dried, ground into powder, and incorporated into the diets of mice with induced chronic colitis [129]. The study found that a diet enriched with 1% bronze tomato fruit powder promoted a change in microbiota composition, moderately inhibiting inflammatory responses in the mice and thus reducing intestinal damage caused by chronic colitis [129]. This was continued in a follow-up study using the same bronze tomato line, which demonstrated the beneficial effects of this variety of tomato on intestinal inflammation and showed changes in the gut microbiome, especially an increase in *Flavobacterium* and *Lactobacillus* and a reduction in *Oscillospira* [130].

In summary, the composition of the gut microbiome is affected by the consumption of tomatoes, and this has implications for human health. Tomato powder has been shown to significantly increase the diversity and richness of the gut microbiome in mice, preventing a build-up of Gram-negative bacteria that produce hepatotoxic compounds, which can cause liver inflammatory disease and cancer [308,310]. Furthermore, freeze-dried tomatoes high in flavanols and anthocyanins have been shown to alter the composition of the gut microbiome by increasing *Flavobacterium* and *Lactobacillus* and decreasing *Oscillospira* populations, resulting in reduced inflammatory responses in mice and preventing intestinal damage by chronic colitis [129,130]. It is worth noting that most evidence shown to date is from animal studies, and it remains to be seen whether tomato product intervention can be beneficial for conditions associated with gut dysbiosis.

### 4.8. Tomatoes and Exercise Recovery

Exercise and increased muscle activity result in higher amounts of ROS due to increased ATP production and oxygen utilisation [312]. ROS are highly reactive and can damage macromolecules such as proteins, DNA, and lipids. ROS such as peroxides and superoxides can be damaging to cells if concentrations are excessive [313,314]. Regular exercise builds resistance of the body against oxidative stress by upregulating the expression of genes that synthesise antioxidant enzymes such as superoxide dismutase [312].

Antioxidants such as vitamins, terpenoids, and phenolics can inhibit oxidative stress and neutralise ROS [314,315,316]. Antioxidants found naturally in plant tissues, including tomato fruit, can provide the protection against ROS. Previous studies have indicated that lycopene and lycopene metabolites can have a positive effect on recovery from exercise-induced physiological stress [144,317]. Lycopene is known to be the most effective singlet oxygen scavenger, exhibiting quenching rates multiple times greater than any other carotenoid [317,318].

Creatinine phosphokinase (CPK) and lactate dehydrogenase (LDH) are associated with ATP and NADH conversion in muscle cells and can be monitored as markers of muscle damage in individuals undergoing exercise sessions [319]. A study by Tsitsimpikou et al. [319] tested whether the administration of 100 g of tomato juice could improve the recovery of anaerobically trained athletes. The study found that a 2-month administration of tomato juice, post exercise, led to a significant decrease in LDH and CPK levels compared to a carbohydrate supplementation beverage [319].

Another study carried out by Nieman et al. [318] administered a “tomato complex” containing lycopene, phytoene, and phytofluene (T-LPP) to endurance runners for a 4-week period and monitored inflammation, muscle damage, and oxidative stress post exercise and during recovery from a two-hour running session. Myoglobin is an iron- and oxygen-binding protein found in muscle tissue that is translocated to the blood compartment after low-level muscle injury caused by exercising and can be used as a marker for muscle injury [318]. Nieman et al. [318] found a significant increase in plasma carotenoid levels and a reduction in myoglobin, suggesting possible reductions in muscle injury as a result of consuming the “tomato complex” supplement following a 2 h running session.

Tomato products and supplementation with their constituents are suggested to reduce muscle damage caused during anaerobic exercise as well as reduce oxidative stress during aerobic exercise, as shown by Harms-Ringdahl et al. [320]. In their study, 15 healthy and untrained participants engaged in 20 min of aerobic exercise on a bicycle after receiving 150 mL of tomato juice for 5 weeks, followed by 5 weeks without tomato juice, and for the final intervention they received tomato juice for another 5 weeks [320]. The blood samples were collected before and after each intervention, and results showed that tomato juice intake significantly suppressed 8-oxodG (a marker of oxidative damage) levels produced with the physical activity [320]. Therefore, there is evidence that tomato products can reduce oxidative stress and muscle damage caused by physical exertion and can be considered as a workout drink [320].

### 4.9. Tomatoes and the Immune System

Tomatoes and tomato products are suggested to affect the immune system [321], and current literature relates this to their lycopene, β-carotene, and vitamin C content. In a human study, supplementation with tomato products (tomato sauce, tomato puree, and raw tomatoes) providing 8 mg lycopene, 0.5 mg β-carotene, and 11 mg vitamin C for 3 weeks was reported to produce a significant increase in plasma levels of lycopene, β-carotene, and vitamin C; however, only lycopene and vitamin C levels increased in the lymphocyte [322]. Supplementation also reduced ex vivo oxidative damage to the DNA of lymphocytes [322]. How vitamin C can affect the immune system has been reviewed by Van Gorkam et al. who describe that, although the data on the effects of vitamin C on B lymphocytes are limited and inconclusive, vitamin C increases the proliferation of T-lymphocytes and natural killer (NK) cells [323]. T-lymphocytes play a key role in cell-mediated, cytotoxic adaptive immunity, and natural killer (NK) cells provide rapid cytolytic responses to virus-infected cells and tumour cells [324,325]. Few studies in human subjects reported that supplementation with β-carotene stimulates the proliferation of lymphocytes [326,327] and enhances the lytic activity of NK cells [5,327]. A study carried out by Watzl et al. [321] supplemented human subjects with tomato juice and carrot juice—both of which are known for their high β-carotene content. This study found that supplementation with the juices significantly increased lymphocyte proliferation and enhanced the lytic activity of natural killer cells [321]. No significant differences were observed between the effects of either juice, indicating a similar effect on the immune response, or that other compounds present in both juices resulted in the observed effects [321].

Naringenin is a flavanone (a subclass of flavonoids) that has also been shown to have immune-modulating functions [328,329]. In an in vitro study, Niu and colleagues demonstrated that naringenin could inhibit T cell activity by various mechanisms, such as lowering the secretion of specific T cell cytokines and affecting T cell proliferation [329]. Further investigation revealed that that inhibition of cell proliferation was triggered by delayed degradation of the cyclin-dependent kinase inhibitor p27kip1 and the downregulation of retinoblastoma protein phosphorylation in activated T cells, resulting in a T cell cycle arrest at G0/G1 phase [329]. Other findings indicated that the T cell-suppressive effects could be attributed to the capacity of naringenin to interfere with the interleukin-2/interleukin-2 receptor (IL-2/IL-2R)-mediated signalling pathway and STAT5 phosphorylation in activated T cells [329].

The immune-modulating effects of lycopene have been hypothesised through their antioxidant activity and their effects on lymphocyte proliferation and on improving cell–cell communication [330,331]. A 2017 study tested 40 mice divided into five groups: an ambient air control; a vehicle control group receiving 200 µL of sunflower oil; a group exposed to cigarette smoke; and two groups administered lycopene diluted in sunflower oil (25 or 50 mg/kg/day) prior to cigarette smoke exposure [332]. The 5-day testing period resulted in an increase in the number of lymphocytes in lycopene-treated groups compared to other treatments [332]. This study suggested that the increase in lymphocytes was a result of lycopene activating the adaptive immune response [333], and the latter is known to be vital in pathogenic defence. However, further studies are warranted to fully understand lycopene’s direct impact on the adaptive immune system in humans [334].

As one of the most popular world crops, the tomato has also been considered as an edible vaccine for a wide variety of diseases, including malaria, coronavirus (COVID-19), human papillomavirus infections, human immunodeficiency virus infections, shigellosis, cholera, anthrax, and hepatitis B [97,335,336,337,338,339]. The main objectives of edible vaccines are to democratize preventive vaccination, especially in developing countries, and to better control potential outbreaks such as coronavirus disease. Traditional vaccine development requires more time and high cost, while the development of an edible vaccine in a plant expression system provides an efficient mode of oral delivery and bypasses the assistance of a medical professional to perform injections. It is also economically sustainable, with higher scale production. However, there are several hurdles to overcome, such as the immunogenicity of an oral vaccine, the stability of the vaccine in the gastrointestinal tract, the variability of the expression of antigens in plants, and the effects associated with the consumption of genetically modified plants on health [336,339]. Shchelkunov et al. designed an oral vaccine against hepatitis B and human immunodeficiency viruses using tomato fruits, which was administered to experimental mice [337]. Examination of serum and stool samples of the test animals revealed high levels of HIV- and HBV-specific antibodies [337]. Salyaev et al. investigated the duration of the mucosal immune response in mice after administration of this vaccine in a subsequent study [340]. Results showed a steady increase in the immune response, with a peak observed between 6 and 11 months post-administration followed by a gradual decrease in the levels of antibodies until they became undetectable after 19 months [340].

Evidence from in vitro, animal, and a few human studies describes a significant increase in lycopene and vitamin C content of lymphocytes, improvements in T cell mediated immunity, and the lytic activity of NK cells, and there is also a suggestion of the use of tomato fruit as an edible vaccine. However, even though plant-based vaccines offer a promising alternative, their clinical development remains challenging, and further research is required in human clinical studies [336,339].

### 4.10. Tomatoes and Fertility

Infertility is a disease of the male or female reproductive system characterised by the inability to accomplish a pregnancy following at least 12 months of regular unprotected sexual intercourse [341,342,343]. According to statistics, 48 million couples and 186 million individuals live with infertility globally [341], and male factors account for at least 50% of all infertility cases worldwide [343]. Oxidative stress (OS), which arises from an imbalance between ROS and protective antioxidants, can affect the entire reproductive lifespan of men and women [344] and has been shown to be a major cause of reproductive dysfunction [344,345].

The positive effects of antioxidants in female fertility have also been described [346,347,348,349]. OS has also been recognised as one of the main mediators of female infertility and has been associated with various reproductive pathologies, including endometriosis, preeclampsia, spontaneous abortion, and unexplained infertility [347,348]. Studies have shown the presence of ROS in the ovaries, fallopian tubes, and embryos of women with idiopathic fertility [348]. Additionally, ROS have been shown to play a role in the regulation of ovarian steroid biosynthesis and secretion, primordial follicle recruitment, and ovulation, and they can also affect the fertilisation process and post-fertilisation events, although the underlying molecular mechanisms have not been fully elucidated [346].

The current literature on folate and fertility endpoints indicates that a high intake of folic acid in the preconception period may increase pregnancy success rates. Upadhyaya et al. showed that folate levels in red-ripe tomato fruits could range from 14 to 46 μg/100 g FW [131]. Furthermore, a lack of vitamin C seems to be associated with an increased risk of preeclampsia, and some studies have shown that vitamin supplements could lower the risk of preeclampsia in normal or underweight women [346]. Some studies have demonstrated that oral administration of multivitamins including folic acid and vitamins C, D, and E can increase fertility [346]. Yu et al. reported that β-carotene has a similar antioxidant potential to folic acid and could also improve the oocyte development and maturation and ovarian function in mice [348]. Therefore, there is some indirect evidence on the role that a tomato-enriched diet may play in female fertility; however, to date, no studies have specifically examined the effects of a tomato-enriched diet on OS-related effects on female fertility.

According to research, between 30 and 80% of male infertility cases are caused by OS and a decreased level of seminal total antioxidant capacity [342,345,350]. Evidence shows that the semen from infertile men has a lower antioxidant capacity and high levels of ROS compared to fertile men [345,346]. As a source of antioxidants, tomato’s constituents and their supplement counterparts may be important for reducing OS and improving semen parameters, including sperm concentration, motility, morphology, and fertility rate [341,343,351]. In a human study, tomato soup consumption at 400 g/day significantly increased seminal plasma levels of lycopene, though the effects on plasma antioxidant levels failed to reach significance [352]. As potent antioxidants, the role of carotenoids in fertility has been extensively investigated [345,348,353,354,355]. Williams et al. examined the effect of lactolycopene, a combination of lycopene with whey protein, which protects lycopene from digestion, on sperm quality in a randomised placebo-controlled trial [354]. Findings suggested that a dose of 14 mg/d lactolycopene over the course of 12 weeks improved the sperm motility and morphology in healthy individuals [354]. Another study by Yamamoto et al. reported similar findings regarding lycopene in a study involving three groups of male infertile patients [356]. On a daily basis, the first group was given 190 g of tomato juice (containing 30 mg lycopene, 38 mg vitamin C, and 3 mg vitamin E), the second group received antioxidant capsules (containing vitamin C 600 mg, vitamin E 200 mg, and glutathione 300 mg), and the third group was given the placebo [356]. The consumption of tomato juice over the course of 12 weeks significantly increased the plasma lycopene level and sperm motility compared to the control group [356]. The group that received the antioxidant capsule, however, showed no significant improvement in semen parameters, suggesting that the increase in plasma lycopene seen in the tomato juice group improved male fertility [356].

Research on the polyphenols, flavonoids, and vitamins of tomatoes, including vitamin E, quercetin, and naringenin, indicates that these compounds may also play important roles in the enhancement of semen quality, including sperm concentration, motility, vitality, and structural integrity [139,341,342,345,349,351,357]. Although other findings are conflicting, according to Aitken et al., at high doses quercetin can have adverse effects on spermatozoa [341,358]. Sabetian et al. provided evidence that oral synthetic vitamin E (400 IU/day) for eight weeks could improve semen parameters and pregnancy rates by neutralising free radical activity and protecting cellular membranes of sperm, which are particularly vulnerable to oxidative damage [139,346,350]. Similarly, in vitro studies in rats and boars have reported the protective effects of quercetin and naringenin on semen [359,360]. Moretti et al. reported that quercetin and naringenin can protect spermatozoa by inhibiting lipid peroxidation in human sperm [361]. Vitamin C, a constituent of tomatoes, has been reported to be present in high concentrations in seminal plasma, and it is established that increasing the concentration of vitamin C in seminal plasma protects against DNA damage [345,346]. Greco et al. conducted a trial involving infertile men treated with both vitamin E and vitamin C [362]. After 8 weeks, the levels of DNA damage were significantly reduced in the treatment group (*p* < 0.001). However, vitamin E and C intake did not seem to have a significant effect on major semen parameters [362].

ROS can be detrimental for fertility both in women and men. Tomato constituents as well as the consumption of tomato products have been suggested to play an important role in fertility. However, the fertility related role of tomato products has only been studied in men, and human intervention with tomato products was shown to increase lycopene levels in the seminal fluids of men and improve sperm motility but failed to improve the antioxidant activity. There are some studies that show an increase in antioxidant activity of seminal plasma with vitamin C and naringenin, which are known to be constituents of tomatoes; however, the current literature also suggests that the individual bioactive compounds of tomato may not have the same mechanisms of action in vivo as their food counterparts [129]. This is likely due to the synergistic action of nutrients when consumed in food rather than individual constituents. Overall, the role of tomato products in fertility requires further investigation to confirm the dose and length of time that is likely to be beneficial for infertility issues both in men and women.

Table 3 provides a summary of the main findings of studies that have indicated a beneficial role of tomatoes and their constituents on age-related chronic diseases as well as fertility- and exercise-induced physiological stress.

## 5. Detrimental Effects of Tomatoes

In contrast to the above-mentioned beneficial effects of a tomato-enriched diet, it is also important to report the potentially detrimental effects that tomato-enriched diets can have on human health. Examples of these detrimental effects include heartburn, irritable bowel syndrome, urinary problems, exposure to pollutants (pesticides, soil herbicides, atmospheric gaseous pollutants, and ethylene gas), migraines, body aches related to glycoalkaloids, anaphylactic reactions, lycopenodermia (an orange or red discolouration of the skin), renal calculi, hepatitis A, and *Salmonella* sp. infections [363,364,365,366,367,368,369,370,371,372,373,374].

A cohort study has revealed that the intake of fruits and vegetables with higher levels of pesticide residue contamination has been associated with poorer semen quality and a lower probability of live birth among couples undergoing fertility treatment [370]. Another investigation showed that pesticides residues on tomatoes may cause harmful health effects and constitute a threat particularly to children’s health [369].

Concerning heavy metal toxicity, it is acknowledged that some metals have the capacity to translocate into plant shoots and accumulate in given plant organs, including roots, stems, leaves, and fruits. More specifically, tomatoes grown in contaminated soil constitute a significant health risk due to a higher potential heavy metal uptake and therefore a higher toxicity [372]. From tomato crop in Quito markets (Ecuador), Romero-Estévez and his team highlighted that levels of lead in tomatoes were near or exceeded the threshold value (0.100 mg/kg) from four markets (0.209, 0.162, 0.110, 0.099 mg/kg), suggesting a possible risk of lead toxicity from tomato consumption [372]. Similar studies also underline the importance of monitoring the content of heavy metals in tomatoes due to their ability to accumulate in the human body and the health risks that they can pose after long-term exposure, even with small doses [371]. Not only is the presence of heavy metal a health risk but it can also adversely impact the levels of nutrients in tomatoes, including lycopene and ascorbic acid [371,372,373].

Another risk involves outbreaks of human *Salmonella* infections and hepatitis A. Three outbreaks of *Salmonella* infections associated with eating Roma tomatoes were detected in the United States and Canada in the summer of 2004 [363]. Between 2005 and 2006, multistate outbreaks of *Salmonella* infections were associated with tomatoes in restaurants in the United States [364]. In addition, three other hepatitis A outbreaks were associated with eating semi-dried tomatoes: in Australia in 2009 and in the Netherlands and in France in 2010 [365,367,374].

The health effects of carotenoids in tomatoes and associated supplements have been extensively discussed, especially lycopene [368,375]. Several studies have reported conflicting findings for the effect of lycopene supplementation on cardiovascular risk factors and cancer [375,376]. Lycopene supplementation is contra-indicated for patients on blood thinners and blood-pressure-lowering medications due to its anti-platelet effect [377,378,379] as it might increase the risks of bruising and bleeding. Recent studies have provided evidence that β-carotene supplements can increase the risk of lung cancer in smokers [73]. Beta-carotene supplements also increased the risk of other cancers [73]. These examples support that the artificial supplements of naturally occurring constituents of tomatoes are likely to work in synergy and that their beneficial properties should not be attributed to one compound alone, although further research would be required to establish these facts [376,380,381,382]. The examples above also provide evidence that most nutrients in fresh fruits and vegetables do not exhibit the same properties as their supplement counterparts that can cause adverse effects [73,182,375,380,381,382]. So far, research has not proven antioxidant supplements to be beneficial in preventing diseases [375].

Consequently, dietary guidelines recommend the regular consumption of fruits and vegetables as part of a balanced diet in order to reap the full benefits of antioxidants in tomatoes. However, one could argue that the bioavailability of beneficial compounds in tomato varies depending on processing and cooking methods, and that one would have to regularly consume tomato in various forms (raw, cooked, boiled, etc.) to access the full range of positive effects [73,380,381,382,383,384,385]. To illustrate, on one hand, thermal processing can significantly increase the bioavailability of carotenoids and phenolics in tomatoes [386,387,388]. In vitro studies have revealed that pulsed electric fields without heat can increase lycopene bioavailability by up to 40%, and when combining it with thermal treatment, by up to 238%, as compared to raw tomato juice [387]. On the other hand, thermal processing can adversely affect the content of other compounds in tomatoes, including water-soluble vitamins and minerals [383,384,385]. Similarly, one could debate whether nutrients from natural unprocessed foods are enough to meet the requirements of human daily intake and confer protective effects against certain diseases, especially when considering the environmental challenges faced by society such as soil erosion and nutrient depletion [389,390], and whether the development of novel genetic engineering and selective breeding techniques could be advantageous [126,391,392,393,394].

## 6. Conclusions

In conclusion, a tomato-rich diet is associated with a diverse range of health benefits, including anticancer properties, reducing the risk of cardiovascular, neurodegenerative, and bowel diseases, and improving skin health, exercise recovery, and immune response. Several factors, including cultivation, processing, the amount consumed, and bioavailability, are likely to influence the overall biological effects of tomatoes seen in the body. The majority of research to date has focused on the biological properties of lycopene. However, there are a number of other bioactive compounds in tomatoes that confer cardiovascular, anticancer, and skin health properties. The synergistic effects of all tomato constituents are likely to outweigh the benefits of tomato’s individual constituents, such as lycopene, and any health benefits of tomatoes should be considered in the wider context of a balanced and healthy diet.

## Figures and Tables

**Table 1:** (**a**) Carotenoids and glycoalkaloids found in tomato ripe fruits. (**b**) Vitamins and polyphenols found in tomato ripe fruits.

**(a)**		**Subclass and Chemical Structure**			**Role in Diseases**
Carotenoids (tetraterpenoids)	Carotenes	α-Carotene [70,71] 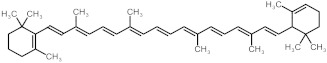			Cardiovascular [72]
		β-Carotene [70] 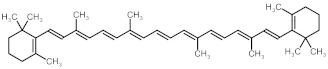			Cardiovascular [72] Cancer [73] Neurodegenerative [74,75]
		Lycopene [70,76] 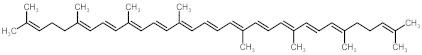			Cancer [77,78,79] Diabetes [80] Cardiovascular [72,81,82] Neurodegenerative [74,75] Skin [83,84,85,86,87]
		Neurosporene [88,89,90] 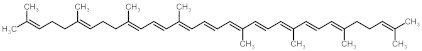			Skin [91]
		Phytoene [89,92] 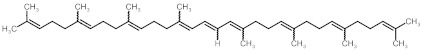			Skin [93]
		Phytofluene [89,94] 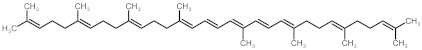			Skin [93]
Glycoalkaloids	Saponins	Tomatine [95,96] 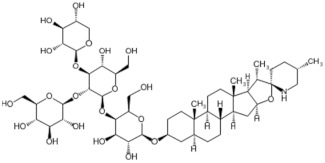			Cancer [97] Cardiovascular [95] Malaria [98]
		Esculeoside A [99,100] 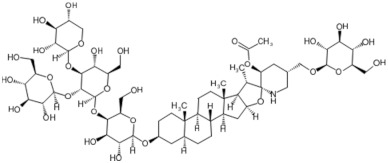			Diabetes [100]
		Lycoperoside H [62,101] 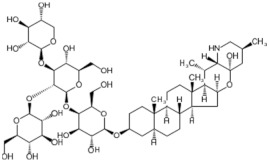			Skin [62] Inflammation [101]
**(b)**					
Polyphenols	Flavonoids	Kaempferol [102,103] 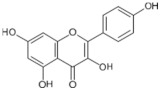	Quercetin [102,104] 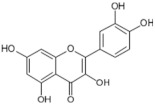	Naringenin [102,105] 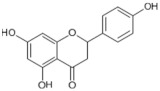	Diabetes [106,107] Skin [108] Neurodegenerative [109]
	Phenolic acids	Caffeic acid [102,110] 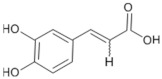	p-Coumaric acid [102,111] 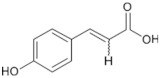	Ferulic acid [102,112] 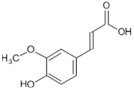	Cancer [113,114,115]
	Stillbenoids	Resveratrol [116,117] 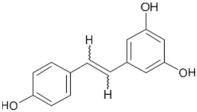			Cancer [118,119] Diabetes [118] Cardiovascular [118] Neurodegenerative [120] Skin [121,122]
	Anthocyanins	Delphinidin [123,124] 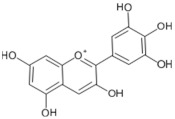	Petunidin [123,125,126] 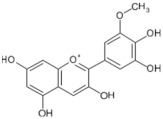		Cancer [127] Cardiovascular [127,128] Neurodegenerative [128] Skin [127] IBD [127,129,130]
Vitamins	B	Folate (vitamin B9) [70,131] 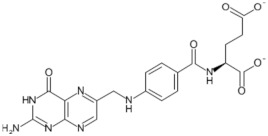			Infertility [132]
	C	Ascorbic acid [70,133] 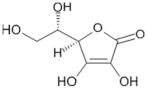			Cancer [134] Diabetes [135] Skin [136,137]
	E	α-Tocopherol [70,138] 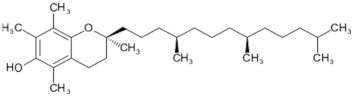			Cancer [134] Diabetes [135] Cardiovascular [139] Infertility [140]
	K	Phylloquinone (vitamin K1) [70] 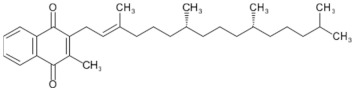			Atherosclerosis [141]

**Table 3:** Main findings of the effects of tomatoes on conditions detailed as part of this review.

Condition	Study Type	Main Findings	References
Hypertension	Double-blind placebo study of grade 1 hypertension patients.	Subjects were administered one Lyc-O-Mato tablet (250 mg capsule consists of 15 mg lycopene (6%), beta carotene (0.15%), phytoene, and phytofluene (1%); and 5 mg vitamin E (2%), phospholipids (15%), and phytosterol (0.6%), suspended in tomato oleoresin oil) per day over an 8-week treatment period. Systolic and diastolic blood pressure was found to decrease following this short-term treatment.	[212]
Atherosclerosis	Randomised, double-blinded, placebo-controlled cross-over study with 90 human subjects selected for normal platelet function. Ex vivo platelet aggregation induced by ADP and collagen were measured.	Study suggested administration of a standardised tomato extract drink (200 mL per day) may have a role in atherosclerosis prevention by reducing platelet activation 3 h after consumption. Significant reductions in ex vivo platelet aggregation were observed 3 h after tomato extract drink consumption.	[225]
Inflammation	Open, prospective, randomised, cross-over, controlled feeding trial.	Participants received either 7 g of raw tomato, 3.5 g of tomato sauce, or 3.5 g of tomato sauce with refined olive oil and 0.25 g of sugar dissolved in water (each per kg of body weight) on four different days with a month interval between each.The day of intervention, blood samples were collected in ethylenediaminetetraacetic acid (EDTA) tubes at baseline (0 h) and 6 h after the supplementation. Results of blood parameter analysis suggested that reduced inflammation was due to decreased LDL-cholesterol levels after tomato supplementation.	[207]
Atopic dermatitis	In vitro study.	Atopic dermatitis is linked to an overproduction of ROS and a decrease in antioxidant defence, as seen in skin biopsy specimens treated with thymodressin (0.1%, 1 mL over 30 days). Tomatoes are a natural source of antioxidants that protect skin cells against ROS.	[304]
Liver inflammatory disease	In vivo study with 49 mice.	Mice supplemented with tomato powder (238.8 mg of lycopene, 10.9 mg of β-carotene, and a trace amount of phytoene per 100 g) in the amount 41.9 g/kg diet. Tomato powder was shown to have a significant increase in microbiome diversity and richness, leading to a reduced production of hepatotoxic compounds such as lipopolysaccharides.	[277]
Neurodegenerative disorders	In vitro study testing human neuroblastoma SH-SY5Y cells.	Cells treated with lycopene (0.2 or 0.5 µM) were monitored for ROS levels, apoptosis, NF-κB activation and Nucling expression, cell viability, mitochondrial membrane potential, and oxygen consumption rate. Lycopene treatment reduced apoptosis by decreasing ROS and inhibited mitochondrial dysfunction and NF-κB target gene Nucling expression in cells.	[252]
Diabetes	In vivo study with 16 mice.	Esculeoside A (main saponin compound in tomatoes) was administered to the mice for 56 days (100 mg/kg). Examination of blood and liver biochemical parameters and liver insulin signalling-related protein expression showed that esculeoside A reduced fasting blood glucose levels and improved glucose tolerance, suggesting it can be a functional supplement for diabetes treatment.	[99]
Exercise-induced physiological stress	Randomised control trial on 15 anaerobically trained athletes of similar age and BMI. Randomised control trial on 15 healthy and untrained participants.	Biochemical evaluation suggested that athlete’s replacement of “usual carbohydrate supplementation beverage” with 100 g tomato juice led to a significant decrease in compounds LDH and CPK related to muscle damage due to anaerobic exercise. Participants engaged in 20 min of aerobic exercise on bicycle after receiving 150 mL of tomato juice for 5 weeks, followed by 5 weeks without tomato juice and tomato juice again for another 5 weeks. The blood samples were collected before and after each intervention, showing that tomato juice intake significantly suppressed 8-oxodG levels produced by aerobic exercise.	[319] [320]
Pathogenic infection	Blinded, randomised, cross-over study.	Enhanced lytic activity of natural killer cells and lymphocyte proliferation observed in human subjects supplemented with 330 mL/day tomato or carrot juice (both high in β-carotene).	[322]
OS and infertility	Randomised experimental study on male infertility patients with poor sperm concentration.	OS, caused by a build-up of ROS, is a main mediator of female infertility. Antioxidants found in tomatoes reduce cellular ROS content. Daily consumption of “one can” of tomato juice (containing 30 g lycopene) was shown to improve sperm motility after seminal parameters were measured every 6 weeks during tomato juice consumption period.	[357]

## Data Availability

Not applicable.
